# Influential Factors and Synergies for Radiation-Gene Therapy on Cancer

**DOI:** 10.1155/2015/313145

**Published:** 2015-12-09

**Authors:** Mei Lin, Junxing Huang, Yujuan Shi, Yanhong Xiao, Ting Guo

**Affiliations:** ^1^Clinical Medical Institute, Taizhou People's Hospital Affiliated to Nantong University, Taizhou, Jiangsu 225300, China; ^2^Medical School, Southeast University, Nanjing, Jiangsu 210009, China

## Abstract

Radiation-gene therapy, a dual anticancer strategy of radiation therapy and gene therapy through connecting radiation-inducible regulatory sequence to therapeutic gene, leading to the gene being induced to express by radiation while radiotherapy is performed and finally resulting in a double synergistic antitumor effect of radiation and gene, has become one of hotspots in the field of cancer treatment in recent years. But under routine dose of radiation, especially in the hypoxia environment of solid tumor, it is difficult for this therapy to achieve desired effect because of low activity of radiation-inducible regulatory elements, low level and transient expression of target gene induced by radiation, inferior target specificity and poor biosecurity, and so on. Based on the problems existing in radiation-gene therapy, many efforts have been devoted to the curative effect improvement of radiation-gene therapy by various means to increase radiation sensitivity or enhance target gene expression and the expression's controllability. Among these synergistic techniques, gene circuit, hypoxic sensitization, and optimization of radiation-induced sequence exhibit a good application potential. This review provides the main influential factors to radiation-gene therapy on cancer and the synergistic techniques to improve the anticancer effect of radiation-gene therapy.

## 1. Introduction

Radiation-gene therapy developed in recent years is a dual anticancer treatment of radiation therapy and gene therapy through coupling therapeutic gene with radiation-inducible regulatory sequence. This therapeutic regiment combines the merits of both gene therapy and radiotherapy. Nevertheless, under routine dose of radiation, especially in the hypoxia environment of solid tumor, desired therapeutic effect is difficult to achieve due to low activity of radiation-inducible regulatory element, low level and transient expression of target genes induced by radiation, inferior target specificity, poor biosecurity, and so on. Based on problems existing in radiation-gene therapy, many efforts have been devoted to the curative effect improvement of radiation-gene therapy by various synergistic techniques to enhance objective gene expression and the expression's regulation or increase radiation sensitivity. Herein, we review the main influential factors of radiation-gene therapy on cancer and the main synergistic techniques to improve anticancer effect of radiation-gene therapy.

## 2. Influential Factors of Radiation-Gene Therapy on Cancer

### 2.1. Influential Factors of Radiation Effect

Radiotherapy is one of the most important anticancer methods which are currently widely used in clinic. According to incomplete statistics, about 70% tumor patients are treated by radiotherapy. However, practices showed that different patients with different tumor types, even with the same type, vary greatly on radiotherapy. Some patients obtained good therapeutic effect while others did not even though they were given a higher dose of irradiation. Their different sensibilities to ionization radiation may be mainly responsible for their distinction curative effects. Radioactive ray itself has energy, which is called radiant energy, and is responsible for radiation's anticancer effect. After cells absorb radiation energy, the DNA of cells is subject to damage by either direct or indirect ionization, and then the cells die. The direct injury is mainly caused by ray which acts on DNA directly, resulting in the DNA strand breaks or DNA crosslinks. In comparison, indirect injury mainly resulted from free radicals produced by ionization of interstitial fluid. These free radicals can work together with biomacromolecules in cells, causing irreversible damage and then leading to cells' death [1]. Hence, all the factors which impact the reactions mentioned above will influence tumor irradiation effect. These factors are mainly associated with oxygen effect, repair capacity to radiation injury, distinct phase of cell cycle, and abnormal expression of genes related to radiation.

Oxygen effect is the chief factor affecting irradiation effect on solid tumors. In the process of ionizing radiation, radiation leads to biotarget molecule damage, forming hydrions and free radicals of biotarget molecules. In the case of oxygen, superoxide radicals may further yield, which further aggravate biomolecule damage. Meanwhile, oxygen can also capture electrons from free radicals of target molecules and prevent target molecules from repairing, causing irreversible damage, which is so-called oxygen effect. Generally, cells in tumor tissue reproduce so quickly that vascular proliferation cannot catch up with them, resulting in oxygen deficit for some cells. These hypoxic cells are resistant to radiation to a great extent. As a rule, their radiosensitivity is only about one-third of normoxic cells. In addition, hypoxic cells are quiescent, relying on energy from anaerobic glycolysis to survive. Although they cannot undergo cell division, they still have a proliferation potential. After irradiation, most of tumor aerobic cells can be killed and tumor grows downwards. But once hypoxia status is improved, these anoxic cells can reproduce and become root for tumor recurrence after radiotherapy. Thus, anoxic cells are mainly responsible for failure of radiotherapy. The amount of hypoxic cells is even considered to be a promising prognostic indicator for malignant tumor [2, 3].

Repair capacity of radiation damage also significantly affects tumor radiation effect. During radiotherapy, radiation directly causes DNA molecules' breakage and cross-linkage. After being irradiated, some damaged DNA molecules will recover with the help of cell repair mechanism, so that some damaged cells can still survive, resulting in radiation effect decrease. Some types of tumor cells, particularly hypoxic cancer cells, have a strong ability to repair damage because they own a larger amount of DNA polymerase *β* (the most important DNA repair enzyme in mammalian). This greatly decreases their sensibility to radiation, resulting in radiotherapy failure. Some researchers connected gene products associated with radiation to impaired DNA to intercept radiation injured DNA from repairing. The findings showed these injured cells were ultimately apoptotic or dead and the therapeutic effect was thus greatly improved, with great decrease of tumor recurrence [4–8].

Abnormal expression of radiation-related genes influences tumor radiation effects, as well. Studies show that p53 gene mutation occurred in over half of all human cancers; tumors with p53 gene mutation have poor radiation sensitivity; but tumor cells transfected with wild-type p53 (wt-p53) genes became significantly more sensitive to radiation, suggesting that p53 gene mutation may cause tumor radiosensitivity decrease. It is currently proved that some other genes are also concerned with irradiation effect, such as p21, p16, ATM, Bcl-2, erbB-2, BHRF1, and PCNA. Once these genes happen to alter, mutation or deletion, tumor radiosensitivity will greatly decline. Among them, the expression level of ATM, Bcl-2, erbB-2, BHRF1, and PCNA is negatively correlated with radiosensitivity, and genetic mutation or deletion of p21 and p16 is correlated as well. In general, genes such as p53, ATM, p16, and p21 have effects on tumor cells mainly through affecting cells' repair ability of radiation injury. Genes including Bcl-2, BHRF1, and erbB-2 affect radiosensitivity by inhibiting tumor cells' apoptosis, whereas p16 and PCNA affect it mainly by regulation of cell cycles [9–16].

In addition, cells in different proliferation cycle have different sensitivity to radiotherapy. Generally, M phase cells are the most sensitive. G1/S and G2/M phase cells are most vulnerable to radiation as chromosome is depolymerized into monomers. Cells in G1 phase are resistant to radiation to some extent, while cells in S phase, especially late S, are the most resistant [17, 18].

### 2.2. Bottleneck of Gene Therapy Development

In recent years, many new gene therapies have been explored for cancer treatment and some of them have exhibited a promising application potential. However, two key problems must be solved to accomplish comprehensive gene therapy against cancer for a desired curative effect. One is how to ensure safe gene delivery into cells with high transfection efficacy. The other is how to make gene expression efficient and controllable.

At present two of major gene transfer vectors, viral vector system and nonviral vector system, have their own advantages and disadvantages. The former, the most efficient up to now, is not regularly employed clinically due to its small gene capacity, poor target specificity, self-immunogenicity, and serious biosafety risk it presents in particular. Despite avoiding major security risks, the latter is greatly inferior to the former in transfection efficiency, and meaningful expression of target genes is hardly available in this system. Currently, lipofection and electroporation are the most widely used methods for nonviral vector transfection with high transfection efficiency. However, liposome is highly cytotoxic and can be quickly cleared by serum* in vivo*, which greatly limits its application. The electroporation method is only suitable for transient or stable expression in cells* in vitro*, but not for transfection* in vivo*. Moreover, a great number of cells will be killed by electric shocks involved. Therefore, how to break through gene transfer bottleneck and ensure therapeutic gene to express efficiently and stably at target sites is a formidable challenge for gene therapy.

Current international research to solve the problems of gene targeting-transfer and expression controllability is mainly focused on three aspects. The first is to rebuild or prepare nonviral gene transfer vector by gene engineering. The second is to covalently link antibody or ligand of tumor cell specific receptor in gene delivery system so that genes can be sent to corresponding tumor cells with the help of similar “biological missiles.” The third is to use a variety of gene regulatory elements to regulate objective gene specific expression in target cells at transcriptional level. Encouragingly, gene transfer vectors based on nanoparticles are emerging, which has become one of the most important achievements in nanobiotechnology research. In addition, it also shows good results in cancer treatment research by coupling specific gene regulatory sequences to objective genes in purpose of regulating the objective genes' expression.

### 2.3. Birth of Tumor Radiation-Gene Therapy and Its Problems

To overcome the limitations existing in respective practices of radiotherapy and gene therapy, Han et al. proposed radiation-gene therapy against malignant tumors in 2006 [19]. To be specific, radiation-inducible regulatory sequence is inserted into therapeutic gene and then transferred into tumor cells. When practicing cancer local radiotherapy, the radiation-inducible regulatory sequence can be activated by radiation and then induce the therapeutic gene to express, resulting in a dual synergistic killing effect of the radiation and the gene on tumor, complementing both advantages, realizing the gene expression controllable [20–22]. That is, radiotherapy can enhance the effect of gene therapy by recombination and integration of DNA, and so forth, inducing and regulating gene expression. On the other hand, gene therapy can improve the effectiveness of radiotherapy by mechanisms related to increasing tumor cells intrinsic radiosensitivity, preventing genes impaired by radiation from repairing, and reducing radiation damage to normal tissue. This therapy has become a new hotspot in the field of cancer treatment, which is a promising therapeutic regiment.

Commonly used radiation-gene therapy techniques principally include combining drug sensitivity gene system with radiation therapy, conjugating tumor suppressor gene to radiotherapy, and connecting immune gene to radiotherapy. Apart from normal radiotherapy role, these technologies, to some extent, make radiation regulating gene expression in space and time realized. However, under routine dose of radiation, especially in hypoxia environment of solid tumor, low activity of radiosensitivity regulatory element, low level and transient expression of target genes induced by radiation, inferior target specificity, and poor biosecurity make desired therapeutic effect difficult to obtain. In order to improve the curative effect of radiation-gene therapy on cancer in the past few years, many synergistic technologies have been explored to enhance objective gene expression and expression's controllability or improve radiation sensitivity against factors affecting the efficacy of radiation-gene therapy. Among them, gene circuit, hypoxic sensitization, and optimization of radiation-induced sequence have been developed considerably and exhibited a promising application potential.

## 3. Synergy Study of Radiation-Gene Therapy

### 3.1. Optimization of Radiation-Induced Sequence

Although most therapeutic genes used inradiation-gene therapy can inhibit tumor cell growth or kill tumor cells, most of them do not have a radiation-inducible property; that is, radiostimulation cannot induce their expression. Theoretically, these therapeutic genes can be coupled with radiation-induced genetic regulatory sequences to confer their radiation-inducible characteristics. For most antitumor genes, this is a key technology to practice tumor radiation-gene therapy.

Early growth response factor 1 (Egr-1) is currently the most studied radiation-inducible promoter, which can be activated by ionizing radiation and then induce its downstream genes to express. This trait has been widely used in radiation-gene therapy study. Some researchers linked Egr-1 promoter sequence to the upstream of therapeutic gene cDNA so that radiation could be used as a switch of priming the genes' transcription to regulate the therapeutic gene expression spatially and temporally. As a result, gene therapy was organically combined with radiotherapy to produce a synergistic antitumor effect [23–26].

A series of deletion analysis to Egr-1 promoter showed that highly conserved repeat sequence CC (A/T) 6GG, named CArG element, is a radiation reaction motif, which is located in the “enhanced” zone of promoter sequence. Only CArG elements (CArG1, CArG2, and CArG3 of E425) located in 5′ promoter region contribute to radiation response of Egr-1, wherein CArG2 responds most strongly. CArG motif is a key for active oxygen substance produced by radiation exposure to activate Egr-1 promoter. CArG elements have many arrangement modes, and different arrangement has different radiation induction. For example, CCATATAAGG (functional CArG element in Egr-1 promoter) has a stronger radiation induction than CCATATTAGG (CArG element in c-fos promoter). Increasing the number of CArG2 elements in promoter can improve specific response to ionizing radiation. Changing CArG core sequence can also greatly influence response to ionizing radiation. Human and murine Egr-1 promoter also contains Sp1 transcription factor, Fos-Jun heterodimer AP-1, and Egr-1 itself putative binding sites, which are all likely to affect Egr-1 response to radiation [27–29].

In order to obtain a better and more specific radiation induction, some researchers have managed to artificially synthesize CArG radiation-induced components. Marples et al. [30] synthetized DNA sequences consisting of four series-wound CArG elements and proved that synthetic CArG sequences had a stronger radiation-induced activity than wild-type Egr-1 promoter, which maybe result from the idea that synthetic CArG enhancer did not contain antagonistic sequence. The synthetic CArG promoter responded to low dose irradiation of 1 Gy, whereas its response reduced when exposed to high doses of radiation at 5 Gy and 10 Gy. Compared with single dose group, irradiation-induced gene expression level in reirradiation group was much higher. With the same accumulated dose, gene expression level of fractionated irradiation group was much more than that of single dose group, indicating that synthetic promoter can be repeatedly activated through fractionated irradiation. It is very vital to obtain high level expression of therapeutic gene in clinic.

To further optimize the ray-inducibility of CArG, Scott et al. [31] constructed promoter vectors containing different number of CArG elements and inserted them into the upstream of reporter gene EGFP and then observed the expression of EGFP after irradiation. They found that the radiation response increased with increasing number (from four to six and then nine) of CArG elements in the promoter. However, it decreased when the number of CArG elements was over 12. Xu et al. [32] synthetized gene CArG elements consisting of nine tandem-repeat CCATATA-AGG combining with reporter gene of luciferase and then transfected them into tumor cells (HeLa, A549, and HepG2) by lipoplast and exposed them to *γ*-ray with different doses. Results showed that the restructured CArG enhancers were able to effectively induce their downstream genes to express at low dose (1 Gy) of radiation, and the expression level got the highest at 3 Gy irradiation. Lung cancer cells transfected with pDNA.CArG.HSV-TK constructed by Zheng et al. [33] showed that their sensitivity to GCV significantly increased and cell survival rate significantly decreased.

As a molecular switch which can confer radiation-induced characteristics for its downstream gene, synthetic radiation-induced promoter containing CArG elements can play an important role in gene therapy. Its induction has a broad spectrum and is valid to polytype tumors. What is more, the radiation response of CArG components does not depend on p53 pathway, which is particularly useful for radiation-gene therapy strategy since more than half of tumors present p53 mutation.

### 3.2. Study on Hypoxic Sensitization

Hypoxia is a ubiquitous problem in solid tumors, which can greatly reduce cells' sensitivity to radiotherapy. Clinically, tumor frequently recurs because hypoxic cells cannot be killed completely. If the unfavorable factor of hypoxia in solid tumors can be overcome, the efficacy of radiation-gene therapy may be greatly improved.

HRE, a hypoxia sensitivity enhancer containing core sequence 5′-(A/G) CGT (G/C) (G/C)-3′, is an important regulatory sequence to mediate hypoxia response. It can specifically bind to hypoxia-inducible factor-1 (HIF-1) to induce its downstream gene to express [34, 35]. Studies indicate that HRE/HIF regulation system exists in both mammalian cells and human tissues, and HIF-1*α* is overexpressed in 68–84% tumors [36]. The fact that HIF-1 possesses the capability of altofrequent expression in tumors stemming from different tissues suggests that HRE can be used to regulate gene therapy in hypoxic environment. Dachs et al. [37] coupled HRE with relevant promoters to construct a HRE/5-FC/CD system. The result demonstrated that CD enzyme activity induced by this system under hypoxic condition was 6.8 times of that under normoxic condition. HRE/CD transgenic cells' sensitivity to 5-FC under hypoxic condition was 5.4 times that under hyperoxic condition. Shibata et al. [38] found that the quantity of HRE also affected the effect of hypoxic sensitization, and they confirmed that combining five copies of HRE with promoter could yield the greatest hypoxic-inducible expression. The 5HRE/hCMV-mp carrier they built made gene expression under hypoxia rise 500-fold.

But simple hypoxia-induced therapy only targets hypoxic cells. In the absence of “bystander” effect, the outer oxygen-rich cells of tumor may escape from destruction. Some researchers have applied hypoxia induction to radiation-gene therapy to improve curative effect. They coupled radiation-sensitive promoter with HRE to form a chimeric promoter HRE/CArG2 which was then inserted into the upstream of therapeutic genes so that therapeutic genes could be activated to express by two mechanisms of hypoxia and ionizing radiation. Therefore, the killing effect in hypoxic area could be enhanced, and the shortcoming that hypoxic tumor was not sensitive to radiation therapy could be overcome and oxygen deficiency could turn into a promoting agent of radiation-gene therapy. The results showed good antitumor activity and therapeutic efficacy [39, 40].

Zhong et al. [41] built a hypoxia/radiation dual sensitive promoter HRE-Egr1 by gene recombination and investigated the expression of double suicide fusion gene yCDglyTK controlled under hypoxia and radiation induction and the killing effect of yCDglyTK/5-FC on nasopharyngeal carcinoma HNE-1 cells. The results demonstrated that yCDglyTK gene expression significantly increased in a cointervention of hypoxia and radiation. The cell survival ratio of hypoxia and radiation therapeutic alliance group was only 4.25%, which was significantly lower than 20.18% of hypoxia induction alone group and 17.41% of ray induction alone group. Wang et al. [42] constructed oncostatin M (OSM) plasmids regulated by hypoxia-radiation dual sensitive promoters. After lung cancer A549 cells were transfected with plasmids and irradiated, the expression of OSM significantly increased under hypoxic condition, which was 3 times as much as that under normal oxygen condition, and the growth of transplanted lung cancer was markedly inhibited and 60% of the tumors completely regressed. The vectors of hypoxia-radiation dual sensitive promoter (HREs-CArG) with HSV-TK gene constructed by Zheng et al. [33] were not only sensitive to radiation exposure but also responded to hypoxia. After hypoxia and irradiation, the downstream HSV-TK gene could be induced to express greatly, which significantly improved the sensitivity of lung cancer cells SPCA1 and A549 to GCV and enhanced the killing efficiency of HSV-TK gene. Compared with pDNA.CArG.MiniCMV.HSV-TK and miniCMV.HSV-TK (control groups), pDNA.HRE.CArG.miniCMV.HSV-TK showed the strongest response to hypoxia and radiation, and the survival ratio of transfected cells after hypoxia and irradiation is minimum.

### 3.3. Synergy of Positive Feedback Loop to Mediate Radiation-Gene Therapy

Gene loop refers to a gene subnetwork composed of different genes which mutually communicate and regulate, having as signal processing functions as a “circuit” to objective gene. This is another hotspot in recent cancer research [43–48]. Through optimizing composition and arrangement of gene circuit elements, the quantity of objective gene expression could be regulated, and the objective gene could be precisely controlled to be expressed or not. In 2004, American “Newsweek” rated gene loop construction as one of the top ten inventions which influence the future. Positive feedback loop, an important model of genetic loops, is a gene subnetwork where proteins coded by specific genes interact with objective genes. It has functions of synchronization, amplification, memory, and positive feedback regulation on objective genes and represents a “waterfall effect” and bimodal distribution pattern. Once initial stimulus reaches a threshold, target gene expression can promptly jump from low level to high level, which moreover can be sustainable. This finding is very significant to realize the controllability of gene expression in gene therapy (e.g., to improve the level and duration of objective gene expression).

p53 response element (pREs) is an enhancer sequence of p53 protein to upregulate its downstream gene expression. By specific binding to it, p53 can induce and enhance target gene expression [49]. Mao et al. [50, 51] brought a positive feedback loop into radiation-gene therapy and successfully constructed wt-p53 protein positive feedback loop mediated by radiation to regulate p53 gene expression. They coupled CArG (E6) which is sensitive to radiation with positive feedback loop composed of pREs (R4) and p53 and constructed recombinant plasmid pE6-R4-p53, a wild-type p53 protein positive feedback gene loop induced by radiation. In this system, wt-p53 expression was improved by using the sensitivity of E6 to radiation. In return, increased p53 reacted on R4 with positive feedback, which further enhanced p53 expression. So cyclically, a positive feedback loop was formed and large amounts of p53 protein were sustainably produced, which is beneficial to overcome radiation resistance caused by p53 mutation and improve the curative effect of radiation-gene therapy. The experimental results* in vitro* and* in vivo* confirmed that radiation-gene therapy mediated by wt-p53 protein positive feedback loop had a strong antitumor effect and this loop can make target gene expression gradually enlarge and persistently express under radiation-induced, and wt-p53 protein is notably increased, accompanied by significant cell cycle arrest and synchronization, resulting in significant increase in the radiation sensitivity of tumor cells and tissues. Immunohistochemical results showed that wt-p53 protein expression (64.8%) of pE6-R4-p53-EGFP/H1299 group was significantly higher than 4.2% of pR4-p53-EGFP/H1299 group, 22.1% of pE6-p53-EGFP/H1299 group, and 0 of H1299 group (no expression). The transplantation tumor growth of pE6-R4-p53-TK/H1299 group was significantly inhibited, whose tumor inhibition ratio (86.41%) was significantly higher than 70.76% of pE6-p53-TK/H1299 group, 35.53% of pR4-p53-TK/H1299 group, and 12.58% of H1299 group. After irradiation, the cells of pE6-R4-p53-EGFP/H1299 group were obviously arrested at G0/G1 (75.13 ± 1.42%), while the arrest rate at S phase decreased (8.63 ± 0.31%). The cell apoptotic rate in each group was higher than that before irradiation, and the apoptotic rate of pE6-R4-p53-EGFP/H1299 group was (23.73 ± 0.21%), which was 5.69, 1.51, and 2.57 times of 3 control groups, respectively. TCD50 (tumor control dose 50%) of xenograft mice was 12.1 Gy, 15.2 Gy, and 19.4 Gy in pE6-R4-p53-EGFP/H1299, pE6-p53-EGFP/H1299, and H1299 (p53-EGFP) group, respectively, and SER (sensitizer enhancement ratio) of pE6-R4-p53-EGFP/H1299 group and pE6-p53-EGFP/H1299 group was 1.6 and 1.28, respectively.

Combining radiation response elements with positive feedback gene loop technology, Kang et al. [52, 53] built a radio-inducible NO synchronized positive feedback genetic circuit using cell-cell signal transduction mechanism and then used this genetic circuit to control the expression of HSV-TK suicide gene. Namely, radiation-inducible c-fos promoter was coupled with inducible nitric oxide synthase (iNOS) gene to build a positive feedback gene circuits (pfos-iNOS/HSV-TK). With rays, iNOS was induced to express, which catalyzed NO synthesis (There are desired substrate and coenzyme in cells). The latter can in turn rapidly activate c-fos promoter by cGMP/GK way to form a positive feedback loop, and the signal molecule NO generated is extremely easy to pass through cell membrane and diffuse freely as a small gaseous lipophilic molecule without charge. Cells with high expression can spur cells with low expression through NO, resulting in gene circuit synchronization and ultimately leading to gene HSV-TK expression synchronization and amplification. In addition, NO itself has a radiation sensitizing property and especially has a significant radiosensitizing effect on mammal hypoxic cells, which can enhance the killing effect of radiation on tumor cells. NO with high level expression itself also has a killing effect on tumor cells, thus forming a triple therapeutic effect of rays, genetic and NO. Moreover, NO has a short half-life (2~4 s) and the effect of NO is relatively localized, which causes no or little damage to distant normal tissues or systemic side effects [54, 55]. The experiments both* in vivo* and* in vitro* exhibited good results. Not only the expression level of target gene was greatly improved, but also the expression synchronization of target gene in transfected cells was realized. Because gene circuit significantly enhanced HSV-TK expression, the sensitivity of tumor cells to HSV-TK/GCV system was significantly improved (increased by 48 times compared with control group), and a much stronger tumor lethal effect was displayed. The inhibitory rate of therapy group was 96%, significantly higher than 75% of GCV group and 82% of control vehicle group. The synchronized positive feedback genetic circuit also has an obvious radiosensitizing effect (TCD50 is 6.16 Gy in A549/pfos-iNOS/TK group, 14.33 Gy in A549/pfos-TK/GFP group, and 15.6 Gy in untransfected A549 group, and SER in A549/pfos-iNOS/TK group and in A549/pfos-TK/GFP group was 2.53 and 1.09, resp., indicating that tumor tissue in A549/pfos-iNOS/TK group was most sensitive to radiation and the synchronized positive feedback genetic circuit greatly increased radiosensitivity of tumor tissue).

Research and development of gene circuit, especially positive feedback loop, provide a new approach to enrich and improve radiation-gene therapy and also offer a new option for other gene therapy.

## 4. Challenges and Prospects

### 4.1. Gene Therapy Combined with Radionuclide Internal Irradiation Therapy

Current radiation-gene therapy mainly employs gene therapy combined with external radiation therapy. The defect is that gene expression induced by external beam radiation is transient, not sustaining. Once the irradiation stops, the objective gene stops expressing, which is not convenient for therapeutic gene to express in tumor continuously. Moreover, external exposure has poor effect on deep tumors and may cause damage to adjacent nontumorous tissues. In contrast, nuclide internal radiation is a continuous low-dose irradiation, but cumulative radiation dose can get high, and it can induce objective gene expression efficiently and continuously. What is more, when marked with specific antibody, receptor, or bioactive peptide, radionuclide can help radiation-gene therapy achieve targeting treatment. Zhao et al. [56] transfected recombinant plasmids Ad.pEgr-1/lacZ into glioma cells* in vivo*. External radiation and intratumoral injection of 125I-UdR both effectively activated Egr-1 promoter and induced downstream gene *β*-galactosidase to express and 125I-UdR concentrated in glioma. This indicated that combining radionuclide internal radiation with gene therapy to practice cancer-targeting treatment is feasible.

### 4.2. Application of Nanogene-Vector in Radiation-Gene Therapy

Lack of suitable gene transfer vectors is not only one of the bottlenecks in clinical gene therapy, but also one of keys to limit research on tumor radiation-gene therapy from advancing. Encouragingly, nanotechnology developed in recent years has offered a new idea to solve gene transfer problem, leading to nanoscale genetic carriers' (referred to as nanocarriers) emergence. Nanocarriers are a class of safe and efficient gene transfer vectors. Their work theory by and large involves the following. Target genes can be coated on nanoparticle surface or embedded inside to form nanogene complex through nanoparticles' surface modification or coupling nanoparticles with specific targeting molecules such as specific ligand and monoclonal antibody. The complex can adhere to cell surface or cell surface antigen or acceptor by electrostatic adsorption or chemical bond and then get into the cells through endocytosis to release the target gene. Thus, the objective of targeted gene therapy or gene transduction is achieved. Compared with traditional carriers, nanocarriers have obvious superiority in gene transferring, including no immunogenicity, nongenotoxicity, or noncytotoxicity, slow release of genes to achieve long-term and stable transgene expression, and so forth and display a promising application potential [57–60]. Tang et al. developed Mn-Zn ferrite magnetic nanoparticle genetic carriers. After combination with P1730OR (mammalian expression vector, encoding *β*-gal, actuated by heat shock protein 70 promoter), Mn-Zn ferrite magnetic nanoparticle genetic carriers can be efficiently transferred into target cells in condition of magnetic transfection. Under the condition of heat shock, *β*-gal expression significantly increased with temperature increasing [61, 62]. In addition to general characteristics of nanoparticles, magnetic nanocarriers have a superparamagnetic. They not only can undertake efficient magnetic transfection and directional movement under an external magnetic field to achieve targeted gene therapy but also can cause magnetic induction heating under an action of external magnetic field to do tumor thermotherapy [63–65].

Based on the above background, it can be conceived that when radionuclide-gene therapy and thermotherapy are combined using magnetic nanoparticles as hyperthermia magnetic medium and gene transfer vector, gene expression will be able to be improved and regulated through radiation promoter while nuclide internal irradiation and each therapy can make their respective advantages complementary to each other; thus a new and effective combination therapy on cancer may be achieved. Meanwhile, coupling HRE and (or) gene circuit with radiation promoter can enhance radiosensitivity of hypoxic tumor cells and improve objective gene expression controllability and thus have better synergistic anticancer effect of radiation-gene therapy.

Radiation-gene therapy may be one of the most promising effective approaches for cancer treatment. However, there are still many problems pending to solve. Although a lot of means have been used to improve this therapy and some improved radiation-gene therapies showed a promising prospect, this research only stays in the stage of cell experiments* in vitro* or animal experiments* in vivo*. It is still far away from clinical trials and practical clinical application. Further study on precise tumor-targeted strategy and on vector system may further promote the development of this therapy. Nanoscale gene transfer vectors have emerged in the field of gene therapy research, showing tremendous superiority to traditional carriers. In the future, it is necessary to further seek more rational and effective therapeutic genes, gene transduction systems, and sensitization techniques on the basis of exploring tumor pathogenesis and growth regulation and to investigate multigene combined with conventional treatment and gene therapy combined with multiple conventional therapies to improve anticancer effect.
